# Prevalence and diversity of enteric pathogens among cholera treatment centre patients with acute diarrhea in Uvira, Democratic Republic of Congo

**DOI:** 10.1186/s12879-020-05454-0

**Published:** 2020-10-09

**Authors:** Camille Williams, Oliver Cumming, Lynn Grignard, Baron Bashige Rumedeka, Jaime Mufitini Saidi, Daniel Grint, Chris Drakeley, Aurelie Jeandron

**Affiliations:** 1 Department of Disease Control, Faculty of Infectious and Tropical Diseases, London School of Hygiene and Tropical Medicine, Keppel Street, London, WC1E 7HT UK; 2 Department of Infection Biology, Faculty of Infectious and Tropical Diseases, London School of Hygiene and Tropical Medicine, London, UK; 3 Ministère de la Santé Publique, Division Provinciale de la Santé Publique, District Sanitaire d’Uvira, Uvira, Sud-Kivu Congo; 4 Department of Infectious Disease Epidemiology, Faculty of Epidemiology and Population Health, London School of Hygiene and Tropical Medicine, London, UK

**Keywords:** Cholera, Diarrhea, Enteric pathogens, Sub-Sahara Africa, Endemic

## Abstract

**Background:**

Cholera remains a major global health challenge. Uvira, in the Democratic Republic of the Congo (DRC), has had endemic cholera since the 1970’s and has been implicated as a possible point of origin for national outbreaks. A previous study among this population, reported a case confirmation rate of 40% by rapid diagnostic test (RDT) among patients at the Uvira Cholera Treatment Centre (CTC). This study considers the prevalence and diversity of 15 enteric pathogens in suspected cholera cases seeking treatment at the Uvira CTC.

**Methods:**

We used the Luminex xTAG® multiplex PCR to test for 15 enteric pathogens, including toxigenic strains of *V. cholerae* in rectal swabs preserved on Whatman FTA Elute cards. Results were interpreted on MAGPIX® and analyzed on the xTAG® Data Analysis Software. Prevalence of enteric pathogens were calculated and pathogen diversity was modelled with a Poisson regression.

**Results:**

Among 269 enrolled CTC patients, PCR detected the presence of toxigenic *Vibrio cholerae* in 38% (103/269) of the patients, which were considered to be cholera cases. These strains were detected as the sole pathogen in 36% (37/103) of these cases. Almost half (45%) of all study participants carried multiple enteric pathogens (two or more). Enterotoxigenic *Escherichia coli* (36%) and *Cryptosporidium* (28%) were the other most common pathogens identified amongst all participants. No pathogen was detected in 16.4% of study participants. Mean number of pathogens was highest amongst boys and girls aged 1–15 years and lowest in women aged 16–81 years. Ninety-three percent of toxigenic *V. cholerae* strains detected by PCR were found in patients having tested positive for *V. cholerae* O1 by RDT.

**Conclusions:**

Our study supports previous results from DRC and other cholera endemic areas in sub-Sahara Africa with less than half of CTC admissions positive for cholera by PCR. More research is required to determine the causes of severe acute diarrhea in these low-resource, endemic areas to optimize treatment measures.

**Trial registration:**

This study is part of the impact evaluation study entitled: “Impact Evaluation of Urban Water Supply Improvements on Cholera and Other Diarrheal Diseases in Uvira, Democratic Republic of Congo” registered on 10 October 2016 at clinicaltrials.gov Identification number: NCT02928341.

## Background

In 2016, infectious diarrheal diseases were estimated to be the eighth leading cause of global mortality, attributing to 1.6 million deaths, and the third leading cause of disability-adjusted life years (DALYs) [1]. Diarrheal morbidity and mortality are concentrated in low and middle-income countries (LMICs) with the highest burden in South Asia and Sub-Sahara Africa (SSA) [2]. Children under five years of age bear most of this burden with diarrheal diseases in early life often leading to malnutrition, growth faltering, cognitive shortcomings, and vitamin deficiencies [2–4].

Diarrheal diseases of infectious origin are predominantly caused by enteric pathogens (including bacteria, viruses, protozoa, and fungi) that are transmitted by oral-fecal routes via ingestion of contaminated food, water or via dirty fingers or fomites, and shed in human and animal feces [5]. Diarrhea caused by the bacterium *Vibrio cholerae* is of particular global concern. In 2015, cholera was endemic in over 47 countries and outbreaks continue to resurface in conflict zones and after natural disasters, such as the outbreaks previously seen in Yemen, South Sudan and Haiti [6, 7]. Severe cholera illness is identified by symptoms of severe dehydration and acute watery diarrhea, defined as three or more loose liquid stools in 24 h, and if untreated, can be fatal within hours of symptom onset [8]. During the first few hours of symptom onset, cholera can be hyper-infectious and person-to-person transmission can increase [9]. However, a large proportion of infections remain asymptomatic and carriers still shed *V. cholerae* but at a lower dose [10]. Cholera particularly affects areas with limited drinking water and sanitation infrastructure [7]. Globally, the highest incidence of cholera is reported in inland Sub-Sahara Africa, particularly in Eastern Democratic Republic of the Congo (DRC) and Western Uganda regions [11, 12].

In 2017, the Global Task Force for Cholera Control (GTFCC) launched a new “Global Roadmap” to reduce cholera mortality by 90% globally and to eliminate cholera in as many as 20 countries by 2030 [8]. This new global strategy led by WHO has three components: (1) early detection and response to outbreaks; (2) a multi-sectoral approach targeting cholera “hotspots”; and (3) effective coordination of support efforts at local and global levels. Comprehensive efforts to control cholera include: ensuring access to safe drinking water and sanitation, and associated hygiene practices; effective treatment for patients, including oral, or intravenous rehydration therapy; and antibiotic treatment for severe cases. In addition to these interventions, safe and effective oral cholera vaccines (OCV) are now available and recommended in many settings [13, 14]; although timely distribution can be challenging due to limited global stockpiles, and the security and logistical challenges inherent to many cholera-affected areas [6, 14, 15].

As part of an ongoing trial (the “Uvira Trial”) to evaluate the impact of improved piped water supply in Uvira on cholera and other diarrheal diseases started in 2016, our study investigates enteric pathogens among suspected cholera patients admitted to a Cholera Treatment Centre (CTC) in Uvira, DRC using a commercial multiplex polymerase chain reaction (PCR) on rectal swabs collected over a nine-month period. The specific objectives are to: (1) quantify the prevalence of enteric pathogens among suspected cholera cases; (2) describe the diversity of enteric pathogens among these patients; and (3) compare confirmation of cholera by PCR and rapid diagnostic test (RDT) methods.

## Methods

### Study setting

Uvira is a city within the province of South Kivu on the eastern side of the DRC along the shores of Lake Tanganyika in the African Great Lakes region with an estimated 233,000 inhabitants in 2017 [16]. Decades of protracted conflict resulting in population displacement combined with limited public health infrastructure have hampered national and international efforts for cholera control in eastern DRC [17]. The African Great Lakes region has been implicated in the spread of outbreaks to other parts of the country and Uvira, in particular, has had endemic cholera since the 1970s [18]. The only CTC in Uvira is located within the district hospital to provide appropriate free treatment for all patients presenting acute diarrhea, defined as: three or more loose/water stools within 24 h [19]. All cases admitted are reported as cholera cases in the National Health Information System (NHIS) and are treated by national protocol for suspected cholera, receiving rehydration therapy and, occasionally, broad-spectrum antibiotics, zinc, or albendazole [19]. A recent linked study to confirm cholera cases by RDT among patients at the Uvira CTC found that only 40% tested positive [19].

### Sampling strategy and data/sample collection

The sampling and recruitment procedures have been described previously [19]. In brief, basic demographic and clinical information for each participant were extracted from CTC patient records, including: age, sex, location of residence, date of admission, date of discharge or transfer, symptoms, and whether the patient received antibiotics prior to rectal swab collection and, if so, the number of hours between first dose and the rectal swab collection. All data were anonymized and entered onto a password-protected tablet by trained researchers and then uploaded to a secure Open Data Kit (ODK) server, and the data then deleted from the tablet.

A trained laboratory technician collected a rectal swab for all new, consenting patients in the morning, seven days a week. The rectal swab was then immediately eluted in 1 ml sterile physiological water for approximately 10 s and lightly shaken before a part is transferred to alkaline peptone water (APW) for an enrichment stage of 6 h before Crystal VC RDT testing (Crystal VC, Span Diagnostics, Surat, India). In parallel, four drops of physiological water (each approximately 40 μl) were preserved on Whatman FTA Elute cards (GE Life Sciences, Piscataway, NJ USA). Once dry, the cards were stored at room temperature in an individual zipped plastic pouch containing a 1 g packet of desiccant silica gel. Previous studies evaluated Crystal VC RDT sensitivity and specificity to be 92 and 91%, respectively [20] and confirmed the effectiveness of Whatman FTA Elute cards (GE Life Sciences, NJ USA) for preservation of nucleic acid for future multiplex PCR analysis [21]. The preserved samples were shipped to the London School of Hygiene and Tropical Medicine for laboratory analysis under the UN3373 infectious substance EXEMPT regulations and under a Material Transfer Agreement (MTA) with the Ministry of Health for DRC.

### Laboratory analysis: DNA extraction and PCR

The Luminex xTAG® Gastrointestinal Pathogen Panel (GPP) was used to analyze the FTA Elute cards for the presence of 15 enteric pathogens: adenovirus, *Campylobacter, Clostridium difficile, Cryptosporidium, Entamoeba histolytica, Escherichia coli* O157, Enterotoxigenic *Escherichia coli* (ETEC) LT/ST, *Giardia,* norovirus GI/GII, rotavirus A, *Salmonella,* shiga toxin-producing *Escherichia coli* (STEC), *Shigella,* toxigenic *Vibrio cholerae,* and *Yersinia enterocolitica* (Luminex Corporation, Austin TX, USA) [22]. The GPP kit is a validated [23–25] commercial real-time PCR assay for the stool-based detection of these pathogens and has been used in various settings [26, 27].

Nucleic acid was extracted from the Whatman Elute Cards using the GE Healthcare Life Sciences protocol (NJ USA) [28]. We added one μL of bacteriophage MS2, the internal control provided by xTAG® GPP to each sample during the wash stage of extraction process, as advised by Luminex®. PCR amplification and hybridization were conducted according to the supplier’s protocol (Luminex Corporation, Austin TX, USA). Results were interpreted on MAGPIX® and analyzed on the xTAG® Data Analysis Software (Luminex Corporation, Austin TX, USA).

### Statistical analysis

All statistical analyses were conducted in STATA version 15 (Stata Corporation, College Station, TX, USA).

The prevalence, and corresponding 95% confidence intervals, for detected enteric pathogens were calculated and stratified by a demographic group, combining both sex and age groups. Poisson regression for count data was used to describe the diversity of enteric pathogens stratified by the demographic group. Robust standard errors were used to control for minor variations from underlying assumptions [29]. The deviance χ^2^ and Pearson χ^2^ goodness of fit tests were used to determine whether data fitted a Poisson distribution. The incident rate ratios (IRR) were recorded along with corresponding *p*-values for a two degrees-of-freedom test. Univariate logistic regression was used to determine if the age and sex demographic group was associated with *V. cholerae* detection. Antibiotic treatment prior to sample collection was identified as a potential source of bias a priori as a single dose of antibiotics can be absorbed in the body in as few as 3 h [30] and, therefore, included in both Poisson and logistic regressions to assess if antibiotic administration influenced the statistical analysis.

Crystal® VC RDT (Span Diagnostics, Surat, India) detects O1/O139 antigens of *V. cholerae* strains whereas the Luminex xTAG® GPP targets the *ctx* gene coding for the cholera toxin, therefore detecting toxigenic *V. cholerae* strains. Discordant pairs for cholera RDT and multiplex-PCR with elute cards were identified. The McNemar χ^2^ test was used to test for marginal homogeneity between these two diagnostic methods.

There were no missing data for any statistical analyses. The corresponding anonymized raw data can be found in Additional File 1.

## Results

### Characteristics and demographics of study participants

Between 24 September 2017 and 09 July 2018, 269 patients admitted to the CTC were enrolled into the study. No patients were enrolled into the study between 11 December 2017 and 11 February 2018 due to a shortage of supplies. Sixty-three percent of study participants were over the age of 15. Sex was evenly distributed amongst study participants (female *n* = 132; and male *n* = 137). Among the participants, 27 (11%) had received antibiotics with a mean time between treatment and sample collection of 6.26 h. Antibiotic treatment was not associated with either the mean number of pathogens present (*p* = 0.2) or *V. cholerae* detection (*p* = 0.3).

### Prevalence and diversity of enteric pathogens

**Fig. 1:**
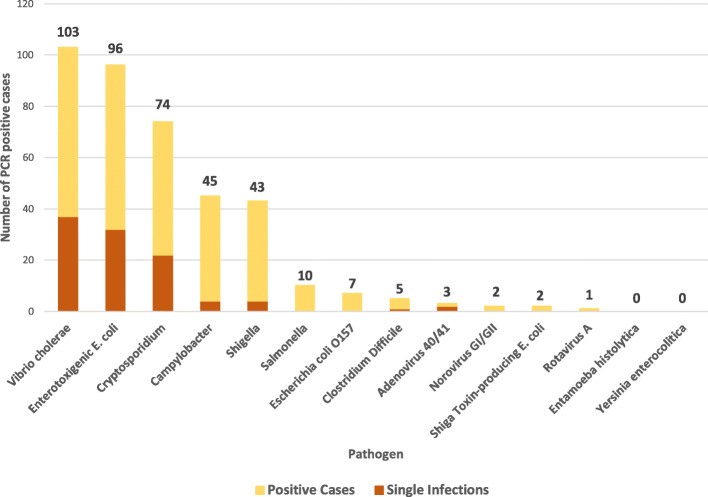
Frequency of specific enteric pathogens detection among enrolled CTC patients

Multiplex-PCR detected 37% (100/269) of all samples were positive for a single pathogen only. Amongst participants with only a single pathogen detected, 37 were V. cholerae, 32 were ETEC, and 22 were *Cryptosporidium*. The distribution of single pathogen detection is shown in Fig. 1. Amongst all study participants, 38% (103/269) were positive for toxigenic *V. cholerae* with men 16–81 years being the demographic group with the highest prevalence at 52%. The other most common enteric pathogens detected were *Cryptosporidium* (28%, 74/269), Enterotoxigenic *Escherichia coli* (36%, 96/269), *Shigella* (16%, 43/269), and *Campylobacter* (17%, 45/269). Forty-five percent of participants carried multiple pathogens (two or more).

Girls 1–15 years had the highest prevalence of carrying multiple pathogens and a fifth of all boys 1–15 years carried three pathogens. Our study detected one boy aged 1–15 years who tested positive for rotavirus. Our study could not detect any enteric pathogen for 16% of the study population. Table 1 refers to the prevalence with 95% confidence intervals of individual pathogens stratified by the demographic group.

**Table 1:** Prevalence of enteric pathogens by age and sex demographic group

Enteric Pathogen	Prevalence (95% Confidence Interval)				
	Total enrolled	Boys 1–15 years	Men 16–81 years	Girls 1–15 years	Women 16–81 years
Total Samples	269	54	83	47	85
At least one pathogen positive	83.6% (78.7–87.6)	85.2% (72.7–92.5)	83.1% (73.3–89.8)	89.4% (76.4–95.6)	80.0% (70.0–87.3)
**Bacteria**	73.9% (68.4–78.9)	68.5% (54.7–79.7)	75.9% (65.4–84.0)	78.7% (64.4–88.3)	72.9% (62.4–81.4)
*Toxigenic Vibrio cholerae*	38.3% (32.6–44.3)	25.9% (15.8–39.5)	51.8% (41.0–62.5)	38.3% (25.3–53.2)	32.9% (23.7–43.7)
Enterotoxigenic *E. coli*	35.7% (30.2–41.6)	40.7% (28.3–54.5)	30.1% (21.1–41.0)	38.3% (25.3–53.2)	36.5% (26.8–47.3)
*Campylobacter*	16.7% (12.7–21.7)	27.8% (17.3–41.4)	10.8% (5.7–19.7)	29.8% (18.3–44.6)	8.2% (3.9–16.4)
*Shigella*	16.0% (12.1–20.9)	24.1% (14.3–37.5)	14.5% (8.3–23.9)	23.4% (13.2–37.9)	8.2% (3.9–16.4)
*Salmonella*	3.7% (2.0–6.8)	3.7% (0.9–14.0)	2.4% (0.6–9.4)	4.3% (1.0–16.0)	4.7% (1.8–12.0)
*Escherichia coli* O157	2.5% (1.3–5.4)	1.9% (0.2–12.5)	2.5% (0.6–9.5)	6.4% (2.0–18.5)	1.2% (0.1–8.1)
*Clostridium difficile*	1.9% (0.8–4.4)	1.9% (0.2–12.5)	–	2.1% (0.3–14.3)	3.5% (1.1–10.5)
Shiga Toxin-producing E. coli	0.7% (0.2–2.9)	–	–	2.1% (0.3–14.3)	1.2% (0.02–8.1)
*Yersinia enterocolitica*	–	–	–	–	–
**Viruses**	2.2% (1.0–4.9)	5.6% (1.8–16.2)	–	2.1% (0.3–14.3)	2.4% (0.6–9.1)
Adenovirus 40/41	1.1% (0.4–3.4)	1.8% (0.2–12.5)	–	2.1% (0.3–14.3)	1.2% (0.2–8.1)
Norovirus GI/GII	0.7% (0.2–2.9)	18.5% (0.2–12.5)	–	–	1.2% (0.2–8.1)
Rotavirus A	0.4% (0.1–2.6)	1.9% (0.2–12.5)	–	–	–
**Parasites**	27.5% (22.5–33.2)	35.2% (23.5–49.0)	26.5% (18.0–37.2)	29.8% (18.3–44.6)	22.4% (14.6–32.6)
*Cryptosporidium*	27.5% (22.5–33.2)	35.2% (23.5–49.0)	26.5% (18.0–37.2)	29.8% (18.3–44.6)	22.4% (14.6–32.6)
*Entamoeba histolytica*	–	–	–	–	–
**Pathogen Diversity**					
*No pathogens*	16.4% (12.4–21.3)	14.8% (7.5–27.3)	16.9% (10.2–26.7)	10.6% (4.4–23.6)	20.0% (12.7–30.0)
*Single pathogen*	38.3% (32.6–44.3)	35.2% (23.5–49.0)	41.0% (30.8–52.0)	21.3% (11.6–35.6)	47.1% (36.6–57.8)
*2 pathogens*	25.6% (20.8–31.2)	18.5% (10.1–31.5)	26.5% (18.0–37.2)	36.2% (23.5–51.1)	23.5% (15.6–33.8)
*3 pathogens*	14.9% (11.1–19.7)	22.2% (12.9–35.5)	12.0% (6.5–21.1)	23.5% (13.2–37.9)	8.2% (3.9–16.4)
*4 pathogens*	3.7% (2.0–6.8)	7.4% (2.7–18.5)	3.6% (1.2–10.8)	6.4% (2.0–18.5)	–
*5 pathogens*	0.7% (0.2–2.9)	–	–	2.1% (0.3–1.4)	1.2% (0.2–8.1)
*6 pathogens*	–	–	–	–	–
*7 pathogens*	–	–	–	–	–
*8 pathogens*	0.4% (0.1–2.6)	1.9% (0.2–12.5)	–	–	–

Our study participants carried a mean of 1.6 pathogens per patient and up to eight different pathogens were detected in a single patient (Interquartile range = 1). Boys and girls 1–15 years carried a greater number of pathogens than adults. There was some evidence indicating boys 1–15 years carried 1.47 (95%CI: 1.13–1.92; *p* = 0.004) times the number of pathogens than women 16–81 years. Likewise, girls 1–15 years carried 1.6 (95%CI: 1.27–2.02; *p* < 0.001) times the number of pathogens than adult women. Table 2 presents the mean number of pathogens by demographic groups.

**Table 2:** Mean number of pathogens detected among enrolled CTC patients, stratified by age and sex

*Count Data Model*	Estimated No. of Pathogens Carried	Standard Deviation	Unadjusted IRR* (95% CI)	*p* Value
*Total*	1.56			
*All males*	1.60	1.23	1.05 (0.88–1.26)	0.35
*Boys 1–15 years*	1.83	1.46	1.47 (1.13–1.92)	0.004
*Men 16–81 years*	1.45	1.03	1.16 (0.93–1.45)	0.19
*All females*	1.50	1.12	Reference**	
*Girls 1–15 years*	2.0	0.95	1.60 (1.27–2.02)	< 0.001
*Women 16–81 years*	1.52	1.09	Reference	

*denotes the incident rate ratio;

**All females are the reference group for all males as sex is a separate variable to the age group

### Comparison of cholera detection by stool-based multiplex-PCR and Crystal VC RDT

Of the 269 study participants, 17 (6%) were RDT-positive for *V. cholerae* O1 but negative for toxigenic *V. cholerae* via PCR and inversely, 19 (7%) were positive via PCR but RDT-negative. There was no evidence suggesting a difference in homogeneity (McNemar’s χ^2^ *p* = 0.74).

## Discussion

All study participants were admitted to the Uvira CTC with suspected cholera, as defined by acute diarrhea, but only 38% (103/269) were positive for *V. cholerae* by rectal swab-based multiplex-PCR analysis. Among the participants, 11 different non-cholera enteric pathogens were detected; of which, ETEC (36%) and *Cryptosporidium* (28%) were the two most prevalent. Eighty-three percent of participants had at least one of the 15 assessed pathogens and almost half (45%, 122/269) were carriers for multiple pathogens i.e. two or more enteric pathogens. Up to eight different pathogens were detected in a single patient. Pathogen diversity was highest among male and female children (age 1–15 years) and lowest among adult women (age 16–81 years).

Our results on cholera confirmation are similar to those of the multi-site AFRICHOL study [31] and suggest that cholera burden in the DRC may be lower than previously estimated. Our PCR results with regard to cholera detection are comparable to results obtained using a commercial RDT among a similar study population [19]. Despite finding no evidence for a significant difference between these two methods (McNemar’s test *p* = 0.7), 7% of study participants were PCR positive but RDT negative and 6% were RDT positive and PCR negative. This could be explained by the performance of the two tests as neither are a gold standard; xTAG GPP methods have 100% specificity but do not have a validated sensitivity (Luminex Corporation, Austin TX, USA) and Crystal VC RDT has a cholera detection sensitivity and specificity of 93 and 91% respectively (Crystal VC, Span Diagnostics, Surat, India). The discrepancies between the PCR and RDT results could also partly be due to the detection of non-toxigenic *V. cholerae* O1 by RDT, although, the proportion of these strains is estimated to be less than 5% in similar contexts [20]. Discrepancies may also be caused by the detection of toxigenic non-O1/O139 *V. cholerae* by PCR. However, the likelihood of having toxigenic strains of serogroups other than O1 or O139, even though they have been implicated as a cause of cholera-like outbreaks [32, 33], is low and has not been reported in a similar epidemiological context. The majority of concordant pairs in RDT/PCR results suggests nonetheless that the *V. cholerae* strains detected were toxigenic O1.

A recent multi-country study for the etiology of moderate to severe diarrhea in children under five years showed the proportion of diarrheal disease attributable to *V. cholerae* increased as age increased [2, 34]. Another study for the change in enteric pathogen prevalence during floods showed the prevalence of toxigenic *V. cholerae* was higher in adults than children [35]. In our own study, we found a higher proportion of *V. cholerae* pathogens in adults than in children, and children carried more pathogens than adults. Current literature also suggests that enteric infections can be asymptomatic as seen in the re-analysis of the GEMS case-control study [34, 36] Infections with *Campylobacter, Cryptosporidium,* and ETEC were often not associated with diarrhea in children under five, and the same study indicates diarrhea could be explained by the combined presence of multiple pathogens or that the severity of a diarrheal illness increases when multiple pathogens are present [34, 36, 37]. In the re-analysis of the MAL-ED birth-cohort study, clinical presentation of illness was also modified when a child had co-infections [38]. Furthermore, both ETEC and *Shigella* have been shown to have long-term growth deficits and *Cryptosporidium* can affect growth even in asymptomatic people [38, 39]. Our results show a mean number of nearly 1.6 pathogens in participants of all ages and all considered as suspected cholera cases. Regardless of causality, these pathogens potentially have positive associations with each other and may increase the severity of diarrheal symptoms.

ETEC and *V. cholerae* represented the majority of detected pathogens in our participants. Both cause profuse watery diarrhea and severe dehydration and are often mis-diagnosed in the absence of laboratory diagnosis [35]. The pathogens included in this study share fecal-oral transmission pathways, either water-borne or water-washed [40] and continued improvements on WASH infrastructure and access to safe drinking water could reduce the main transmission routes of many of these pathogens. Only one case of rotavirus was detected in our samples, although the burden attributed to rotavirus in LMICs is reportedly high [1]. Two possible reasons for this are: rotavirus most commonly infects children under the age of five, an age group for which we had few participants; preservation on FTA Elute cards and storage may also have had more detrimental effect on viral DNA/RNA than on other pathogen’s nucleic acids.

### Limitations

There are a number of limitations to this study. First, our study only included people admitted to the CTC and, therefore, does not capture symptomatic cholera cases who do not seek care or asymptomatic carriers [10, 41]. Secondly, we opted to use rectal swabs for sampling, instead of direct stool, to mitigate the high risk of cross-contamination or contact with chlorine. A previous study found rectal swabs had similar detection rates when compared to direct stool samples during PCR analysis [42]. Storing samples on FTA elute cards for the Luminex PCR assay has yet to be validated; although, multiple studies have described FTA elute cards as a comparable preservation method when compared to fresh and frozen samples [21, 43]. Literature also suggests there are no signs of nucleic acid degradation with short-term storage on elute cards; however prolonged preservation, humidity, and high temperatures may negatively impact nucleic acid preservation, particularly with RNA [21, 43–45]. In our study, viral pathogens were lower than expected, which could be attributed to viral nucleic acid degradation. This may explain why rotavirus was detected in only one sample. Therefore, negative results should be interpreted with caution. *Giardia* was found by the manufacturer to cross-react with other organisms in stool samples and accurate detection would have required a follow-up identification method for all positive samples, which was not possible in our study. Therefore, *Giardia* detections results were not included in the statistical analysis. No fungal pathogens are targeted by the Luminex xTAG GPP. Luminex xTAG® GPP detects toxigenic *V. cholerae* strains while Crystal® VC RDT detects O1/O139 antigens of *V. cholerae* strains. Therefore, we were not able to conclude whether testing performance or variety of *V. cholerae* strains are leading to discordant results between both methods and we may not be detecting non-toxigenic non-O1/non-O139 strains that can be responsible for diarrhea. Finally, to determine the pathogens truly associated with acute diarrheal disease amongst admitted CTC patients would have required a different study design and the use of quantitative PCR methods to assess the etiological quantity of detected pathogens [46]; however, in the circumstance of acute diarrhea, single pathogen detection is likely sufficient to determine enteric infections [47] as symptomatic individuals typically shed a higher quantity of pathogens [47–49].

## Conclusions

Our finding that more than half the patients admitted to a CTC were not positive for cholera by stool-based PCR and RDT assays are consistent with previous results in DRC and other cholera endemic areas of sub-Sahara Africa. However, the prevalence of the assessed enteric pathogens was high among patients admitted to the Uvira CTC with 84% having at least one of the 15 assessed pathogens, and 45% carrying multiple pathogens. Our findings lend support to the current strategy of the DRC Ministry of Health for the prevention and control of cholera, which targets “hotspot” areas such as North and South Kivu, and encourages future actions to continue with a comprehensive strategy that includes both improved water, sanitation and hygiene and effective and timely treatment of cases.

## Supplementary information

**Additional file 1.**

## Data Availability

All data generated and analyzed during this study are included in the published manuscript and supplementary information files.

## Abbreviations

APW: alkaline peptone water
CTC: Cholera Treatment Centre
DALYs: Disability-adjusted life years
DRC: The Democratic Republic of the Congo
ETEC: Enterotoxigenic *Escherichia coli*
GPP: Gastrointestinal Pathogen Panel
GTFCC: Global Task Force for Cholera Control
LMICs: Low and middle-income countries
LSHTM: London School of Hygiene and Tropical Medicine
MTA: Material Transfer Agreement
NHIS: National Health Information System
OCV: Oral cholera vaccines
ODK: Open Data Kit
PCR: Polymerase chain reaction
PIS: Participant information sheet
RDT: Rapid diagnostic test
SSA: Sub-Sahara Africa
STEC: Shiga toxin-producing *Escherichia coli*
